# Brief Exposure to Novel or Enriched Environments Reduces Sucrose Cue-Reactivity and Consumption in Rats after 1 or 30 Days of Forced Abstinence from Self-Administration

**DOI:** 10.1371/journal.pone.0054164

**Published:** 2013-01-16

**Authors:** Jeffrey W. Grimm, Rachel Weber, Jesse Barnes, Jon Koerber, Kylan Dorsey, Edwin Glueck

**Affiliations:** Department of Psychology and Program in Behavioral Neuroscience, Western Washington University, Bellingham, Washington, United States of America; Radboud University, The Netherlands

## Abstract

Environmental enrichment (EE) reduces drug and sucrose cue-reactivity in rats. In a previous study we reported that 1 month of EE (large cage, toys, and social cohorts) significantly reduced sucrose cue-reactivity. In the present study, we examined whether overnight (22 h) EE would be as effective. We also examined whether social enrichment (SE), enrichment alone (SoloEE), or exposure to an alternative environment (AEnv) might account for the EE effect. Rats self-administered 10% sucrose (.2 mL/delivery) in 10 daily 2-h sessions. Sucrose delivery was accompanied by a tone+light cue. Rats were then exposed to enrichment or alternative environment conditions overnight (acute) or for 29 days (chronic). Sucrose cue-reactivity was measured after this period of forced abstinence in a session identical to training, but no sucrose was delivered with the cue. All acute conditions markedly reduced sucrose cue-reactivity after 1 day of forced abstinence compared to single-housed rats in standard vivarium housing (CON). Sucrose consumption was also significantly reduced in all groups but SoloEE in a next-day test. All acute conditions but SE significantly reduced sucrose cue-reactivity when administered just prior to Day 30 of forced abstinence; all reduced sucrose consumption in a next-day test. All chronic conditions except for SE and AEnv significantly reduced sucrose cue-reactivity on the Day 30 test and sucrose consumption in a next day test. For both acute and chronic comparisons, EE manipulations were the most effective at reducing sucrose cue-reactivity and consumption. SoloEE and EE were equally effective at reducing sucrose cue-reactivity and similarly effective at reducing sucrose consumption. This indicates that social interaction is not a necessary condition for reducing sucrose-motivated behaviors. These results may be useful in the development of anti-relapse strategies for drug and food addictions.

## Introduction

Drug abuse continues to contribute to negative health and social outcomes [1], [2]. Attention has recently turned to excessive food consumption (“food abuse”) as obesity rates have doubled in some regions of the US between 1999–2008 [3]. It has been suggested that disordered eating and drug addiction share common neurobehavioral features [4], [5], [6]. Sucrose self-administration by rats provides not only a model of addiction behaviors relevant to understanding drug addiction, but even more specifically food preoccupation behaviors that may contribute to overeating and obesity [7].

We and others have examined various aspects of sucrose seeking and taking behaviors in rats. In our typical procedure, rats acquire self-administration in daily sessions in operant conditioning chambers where lever responding is reinforced with liquid sucrose deliveries that are paired with the presentation of a visual and auditory stimulus. Responding is then tested in the absence of sucrose, but with the sucrose-paired stimulus still available. Rats will respond for delivery of this stimulus, and this response rate increases over a period of forced abstinence from sucrose self-administration [8]. This abstinence-dependent increase in sucrose cue-reactivity (“incubation of craving”) is similar to what has been observed in rats with a history of drug (cocaine, methamphetamine, nicotine, alcohol) self-administration and in humans with a history of cocaine, heroin, or cigarette abuse [7].

In characterizing the incubation of craving effect in rats, we and others have examined the effectiveness of behavioral and pharmacological manipulations to reduce sucrose cue-reactivity [6], [8], [9]. One exceptionally robust manipulation that appeared to block the incubation of sucrose cue-reactivity was one month of environmental enrichment [10]. The effect was strikingly similar in rats with a history of cocaine self-administration [11], [12].

With the overall aim of examining neural substrates of the enrichment effect, the present study was conducted to first parametrically evaluate key components of an enriched environment that lead to decreased sucrose cue-reactivity in rats over a period of forced abstinence from sucrose self-administration. We examined the effects of acute (22 h) vs. chronic (29 days) isolated housing (CON), social enrichment (SE), context enrichment (SoloEE), exposure to an alternative environment (AEnv), or “full” environmental enrichment (EE) on sucrose cue-reactivity after a brief or protracted period (1 or 30 days) of forced abstinence. Sucrose consumption was also measured in all rats the day following cue-reactivity testing.

It was found that while in some cases SE and AEnv reduced sucrose cue-reactivity and consumption, exposure to the EE context consistently produced the greatest decreases in sucrose cue-reactivity and consumption. Acute exposure to these manipulations was in many cases just as, if not more effective than, chronic exposure. It was also found that nearly all of the manipulations that were chronic or administered just prior to the 30^th^ day of forced abstinence blocked the expression of the incubation of sucrose cue-reactivity.

## Materials and Methods

### Subjects

179 male Long-Evans rats (approximately 3.5 months old; 455.1±4.6 g (mean ± standard error of the mean) (SEM)) at start of study; Simonsen-derived, Gilroy, California, USA) bred in the Western Washington University vivarium were housed individually on a 12-h reverse day/night cycle (lights off at 0700) with Purina Mills Inc. Mazuri Rodent Pellets (Gray Summit, MO, USA) and water available *ad libitum* in home cages and in operant conditioning chambers. All training and testing took place between 0900-1500 with cohorts of rats always trained and tested at the same time daily. Rats were weighed each Monday, Wednesday, and Friday for the duration of the experiment. Immediately prior to the training phase, the animals were deprived of water for 17 h to encourage sucrose self-administration on the first day of training. All procedures followed the guidelines outlined in the “Principles of Laboratory Animal Care” (NIH publication no. 86–23) and were approved by the Western Washington University Institutional Animal Care and Use Committee.

### Apparatus

Operant training and testing took place in operant conditioning chambers (30×20×24 cm; Med Associates, St. Albans, VT, USA) containing two levers (one stationary and one retractable), a tone generator, a white stimulus light above the retractable lever, and a red house light on the opposite wall. An infusion pump delivered sucrose into a reward receptacle to the right of the active lever. Operant conditioning chambers were enclosed in sound-attenuating cabinets with ventilation fans.

### Sucrose Self-administration Training

Rats spent 2 h/day for 10 consecutive days in operant conditioning chambers and were allowed to press the retractable (active) lever on a fixed-ratio 1 schedule for a 0.2 ml delivery of 10% sucrose solution into the receptacle to the right of the lever. This response also activated a compound stimulus consisting of a tone (2 kHz, 15 dB over ambient noise) and the white light. The compound stimulus lasted for 5 s and was followed by a 40-s time out, during which presses on the active lever were recorded but had no programmed consequence. A response on the inactive (stationary) lever had no programmed consequence, but presses were recorded. Four infrared photobeams crisscrossed the chamber. The total number of beam breaks was recorded during training and testing. At the end of each training session, rats were returned to home cages.

### Forced-abstinence

The forced-abstinence phase began immediately following the 10^th^ day of the training phase. That day will be referred to as the first day, or “Day 1″, of forced abstinence.

### Treatment Conditions

**Figure 1:**
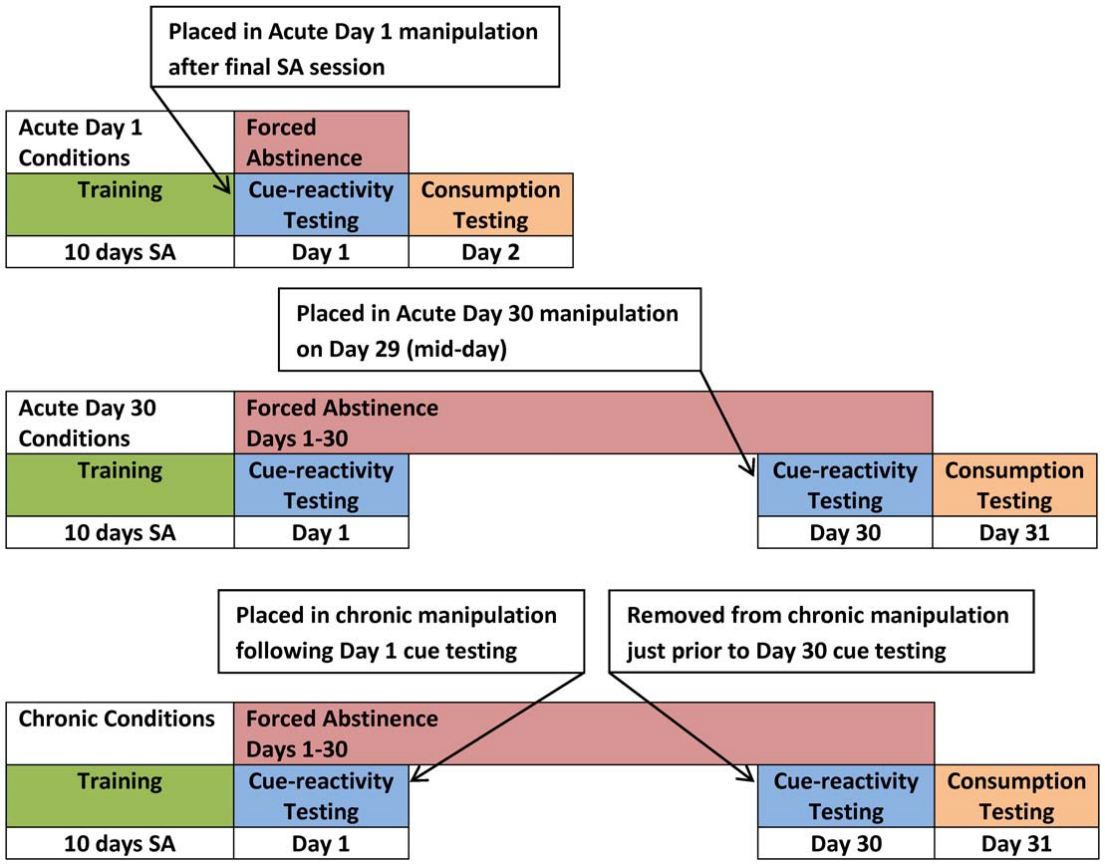
General experimental plan. Following 10 days of sucrose self-administration (SA) rats were moved into acute or chronic manipulations (see Table 1 for manipulations details). All rats were moved back into CON conditions following Day 30 Cue-reactivity testing (or Day 1 Cue-reactivity testing for Acute Day 1 manipulations).

Rats were randomly assigned to treatment conditions following self-administration training. Treatment conditions were either acute or chronic (Figure 1). Acute exposure was 22 h prior to the cue-reactivity test. Chronic exposure was from the afternoon of Day 1 cue-reactivity testing to immediately prior to the Day 30 cue-reactivity test. In addition to the acute and chronic manipulations, there were five treatment conditions: control (CON), social enrichment (SE), environment-only enrichment (SoloEE), environmental enrichment (EE), or alternative environment (AEnv). Details of these conditions are provided in Table 1.

**Table 1:** Treatment Condition Details.

Treatment Condition	Detail
Control (CON)	Continued single housing in standard-size cage on self-contained cage rack
Social Enrichment (SE)	Two rats in double-sized cage on self-contained cage rack
Enrichment Only (SoloEE)	One rat in very large four-level cage with toys (novel toys M,W, F) and shelters (30 cm long x 11 cm diameter P.V.C. pipe and 25 cm diameter plastic rodent “igloo”)
Environmental Enrichment (EE)	Three rats in very large four-level cage with toys (novel toys M,W, F) and shelters (30 cm long x 11 cm diameter P.V.C. pipe and 25 cm diameter plastic rodent “igloo”)
Alternative Environment (AEnv)	Single housing in white plastic cage with wire bar lid placed away from CON cage rack

Note. Cage dimensions (width x depth x height in cm): CON (20×32×20), SE (45×32×20), SoloEE and EE (91×51×102), AEnv (22×43×20).

CON, SE, and AEnv cages were from Lab Products Inc. (Seaford, DE, USA) and the SoloEE/EE cages were from Quality Cage Company (Portland, OR, USA). The rationale for the acute and chronic manipulations was to identify whether relatively brief exposure to enrichment could produce a change in sucrose cue-reactivity. Such an effect has recently been described for cocaine cue-reactivity in rats [11], [13]. The rationale for examination of the effect of acute manipulations after either 1 or 30 days of forced abstinence was to test whether acute manipulations were more or less effective at altering “incubated” sucrose cue-reactivity. Finally, the five treatment conditions were included to examine the potential contribution(s) of social interaction (SE group), an environmentally-enriched (but not socially-enriched) environment (SoloEE), and/or exposure to a context other than the home cage or operant conditioning chamber (AEnv) to the EE effect we reported previously [10].

### Sucrose Cue-reactivity Testing

On Day 1 and Day 30, rats were tested in the operant conditioning chambers for sucrose cue-reactivity (sucrose seeking). This session was identical to the 2-h training procedure, but sucrose was not delivered following a lever response. Following the Day 1 test, rats assigned to a chronic manipulation were placed into those conditions and rats that had just received an acute manipulation were returned to CON housing conditions. Following the Day 30 test, all rats were returned to CON housing conditions.

### Sucrose Consumption Testing

On Day 2 *or* Day 31, rats were returned to the operant conditioning chambers for a sucrose self-administration test (Consumption). The test was to evaluate any persistence of enrichment or novelty effects on motivation to consume sucrose itself. This session was identical to the 2-h training procedure. A separate group of consumption CON rats (n = 11) was run with consumption testing on Day 2. This was the comparison group for all Day 1 and Day 2 behaviors. All other CON rats only had consumption testing on Day 31. This was the comparison group for all Day 30 and 31 behaviors.

### Statistical Analyses

Active lever responding, sucrose deliveries, inactive lever responding, and photobeam breaks during sucrose self-administration training were analyzed using mixed-factor ANOVAs of the 10 days of training (TIME) and the between-group factor of MANIPULATION. There were 14 levels of MANIPULATION as there were 14 distinct groups of rats in the study. This analysis was used to verify that all of these groups received equal training. Acquisition of sucrose self-administration was defined as an average of 20 or more daily sucrose deliveries over the final four days of self-administration training for each rat, and an overall group increase in responding for sucrose over the 10 days of training. Testing data were first analyzed separately for each day of forced abstinence. For the two hour cue-reactivity and consumption tests, the effects of MANIPULATION on each dependent measure (active lever responses, inactive lever responses, photobeam breaks) were evaluated using ANOVA. There were 5 levels of this variable for the Day 1 comparison and 9 levels of this variable for the Day 30 comparison. Two Pearson’s r correlations were subsequently calculated to compare cue-reactivity with consumption responding (see Discussion). To verify incubation of craving in the CON condition, one t-test was calculated to compare CON Day 1 vs. CON Day 30 active lever responding.

To further evaluate abstinent-dependent effects of manipulations on cue-reactivity, all active lever response data was converted to a percent of the average responses of the CON Day 1 and then compared using ANOVA (13 levels of MANIPULATION). Two additional ANOVAs were calculated to examine any enduring effects of acute manipulations experienced prior to Day 1 by comparing the Day 30 and 31 active lever responding of groups that had an acute manipulation prior to the Day 1 cue-reactivity test (5 levels of MANIPULATION, see Discussion).

For all statistical comparisons other than post-hoc tests, p<0.05 was the alpha criterion for statistical significance. ANOVA post-hoc comparisons were made with one-tailed t-tests using Bonferroni family-wise error rate correction-adjusted alpha levels to determine statistical significance. These corrected, more conservative, alphas were used to avoid Type-1 error. ANOVAs and correlations were calculated using SPSS version 19. T-tests were calculated using EXCEL 2010. Group data are presented as means ± SEMs in the text and figures. In general, only the statistics for significant effects and interactions are indicated in the text. For the post-hoc tests, we chose to reduce both Type-1 and Type-2 error by asking specific questions rather than examining all possible differences between groups. First, we compared manipulation groups to the relevant CON condition to determine whether a particular manipulation decreased sucrose seeking or consumption. Next, we compared all manipulations to the EE Acute manipulation (EE Acute Day 1 for final all-groups percent of CON Day 1 comparison) as in all cue-reactivity comparisons EE Acute was ranked as the most effective manipulation at reducing cue-reactivity versus the CON group. We used this approach as we felt that the EE Acute manipulation provided a benchmark to compare the relative importance of the various manipulations that consisted of various components of EE Acute (social enrichment, contextual enrichment, and novelty). In addition, we chose to primarily examine the effects of the various manipulations using between-groups comparisons at Day 1 or Day 30. Therefore, not all data collected (e.g. Day 1 cue-reactivity of rats in the Day 30 acute or chronic conditions) are represented in the Results.

## Results

Of 179 rats that were trained for sucrose self-administration, 7 were removed from the study because they did not meet a minimum response criterion for acquisition of an average of 20 sucrose deliveries/day over the last four days of training. Final group sizes are indicated in Tables 2 and 3.

**Table 2:** Inactive Lever Responding and Photobeam Breaks during Cue-reactivity Testing (mean ± SEM).

TreatmentCondition	n	Inactive LeverResponses	PhotobeamBreaks
Day 1 Testing Groups			
CON	11	7.8±1.8 x	1837.9±155.9 x
EE Acute	15	0.5±0.2 *	638.1±95.2 *
SoloEE Acute	11	0.7±0.3 *	1120.2±372.7
AEnv Acute	9	3.2±2.5	918.7±103.4 *
SE Acute	14	1.4±0.4 *	1038.6±108.1 *
Day 30 Testing Groups			
CON	24	22.1±5.6 x	2387.1±109.1 x
EE Acute	9	1.3±0.6	876.4±170.9 *
SoloEE Acute	8	3.1±1.0	1198.6±138.6 *
EE Chronic	15	4.6±1.1	1155.7±122.5 *
SoloEE Chronic	8	6.4±2.4 x	882.1±149.5 *
AEnv Acute	9	14.2±4.3	1377.9±205.3 x
SE Acute	16	11.8±1.6 x	1781.4±166.3 *x
SE Chronic	14	13.4±2.5 x	1644.6±111.3 *x
AEnv Chronic	9	11.8±2.5 x	1160.9±154.9 *

Note.

* indicates significant difference from CON group for that Day of testing and x indicates significant difference from EE Acute group for that Day of testing, p<0.05.

**Table 3:** Inactive Lever Responding and Photobeam Breaks during Consumption Testing (mean ± SEM).

TreatmentCondition	n	Inactive LeverResponses	PhotobeamBreaks
Day 2 Testing Groups			
CON	11	6.4±2.1	2179.4±231.2 x
EE Acute	15	2.1±0.7	1143.6±72.4 *
SoloEE Acute	11	6.1±3.5	1305.3±139.6 *
AEnv Acute	9	9.3±4.6	1121.0±200.3 *
SE Acute	14	2.4±0.7	1668.3±200.8 x
Day 31 TestingGroups			
CON	24	17.9±4.5 x	2354.2±96.0 x
EE Acute	9	5.9±2.6	1483.4±107.9 *
SoloEE Acute	8	7.4±3.1	1996.0±235.4
EE Chronic	15	2.1±0.7 *	1255.7±148.0 *
SoloEE Chronic	8	4.1±2.0	1014.3±86.2 *
AEnv Acute	9	10.4±4.1	2127.7±258.2
SE Acute	16	10.4±2.0	2138.8±214.9
SE Chronic	14	10.5±1.5	1894.9±117.5 *
AEnv Chronic	9	6.0±2.2	1439.0±110.4 *

Note.

* indicates significant difference from CON group for that Day of testing and x indicates significant difference from EE Acute group for that Day of testing, p<0.05.

All remaining rats acquired sucrose self-administration with active lever responding and sucrose deliveries increasing over the 10 days of training (active lever TIME F(9,1422) = 5.9, p<0.001; infusions TIME F(9,1422) = 39.0, p<0.001) and inactive lever responding decreasing over the 10 days of training (TIME F(9,1422) = 103.0, p<0.001). Locomotor activity also decreased over the 10 days of training (TIME F(9,1422) = 46.3, p<0.001). There were no significant differences between the 14 groups of animals. Average rates of responding on the final day of training were active lever, 166.2±6.1, infusions, 83.1±2.0, inactive lever, 6.1±0.6, and photobeam breaks, 1946.3±38.4.

### Sucrose Cue-reactivity Testing

**Figure 2:**
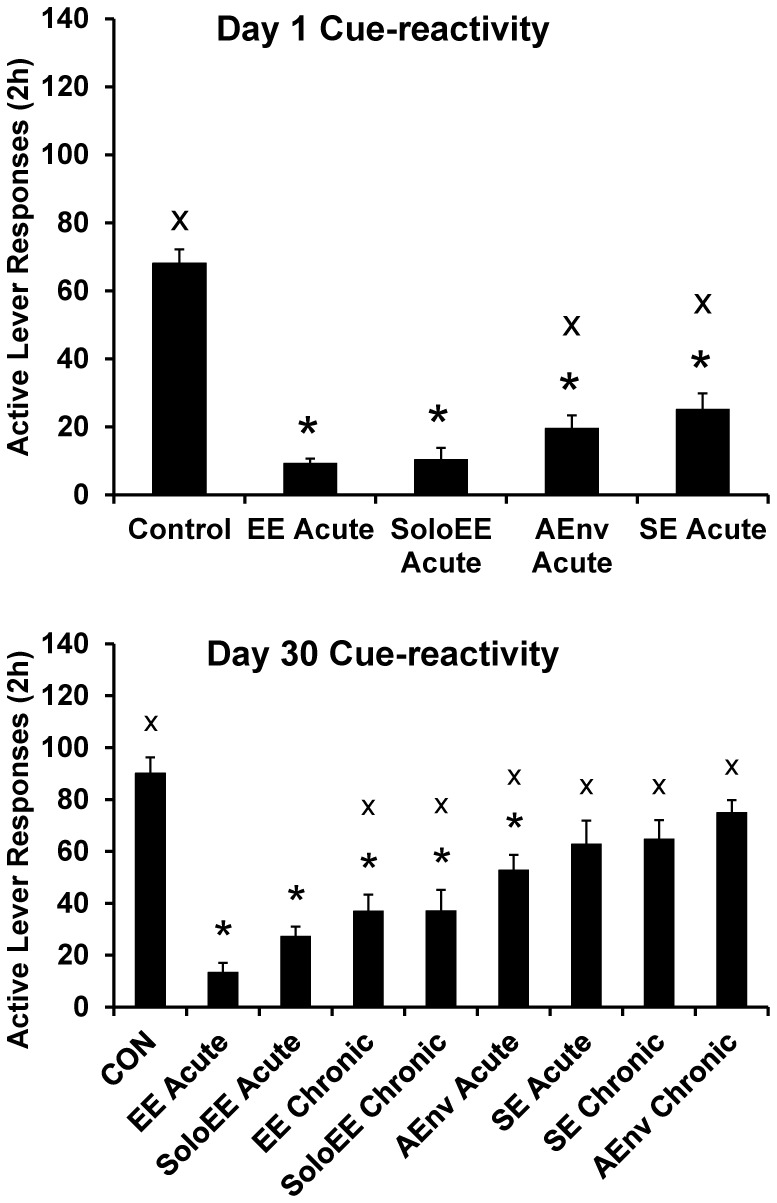
Cue-reactivity after 1 or 30 days of forced-abstinence and an acute or chronic manipulation. Group data are presented ranked by group averages. * indicates significant difference from CON group and x indicates significant difference from EE Acute group, p<0.05.

For Day 1 responding there was a significant effect of MANIPULATION for active lever responses (F(4,55) = 40.8), inactive lever responses (F(4,55) = 6.8), and photobeam breaks (F(4,55) = 5.8), all p<0.01. For Day 30 responding there was a significant effect of MANIPULATION for active lever responses (F(8,103) = 11.8), inactive lever responses (F(8,103) = 3.2), and photobeam breaks (F(8,103) = 14.1), all p<0.01. Active lever responses and selected post hoc tests results are presented in Figure 2. In Figure 2, groups are presented to the right of the CON group ranked from lowest to highest mean response rate. CON Day 30 active lever responding was significantly greater than CON Day 1 t(33) = 2.3, p<0.05) indicating an incubation of craving under control conditions (CON Day 1 vs. CON Day 30 significance not indicated on Figure 2).

The effects of the various manipulations on inactive lever responses and photobeam breaks were fairly similar to their effects on active lever responses. Means ± SEMS of inactive lever responses and photobeam breaks along with post-hoc tests are presented in Table 2.

### Sucrose Consumption Testing

**Figure 3:**
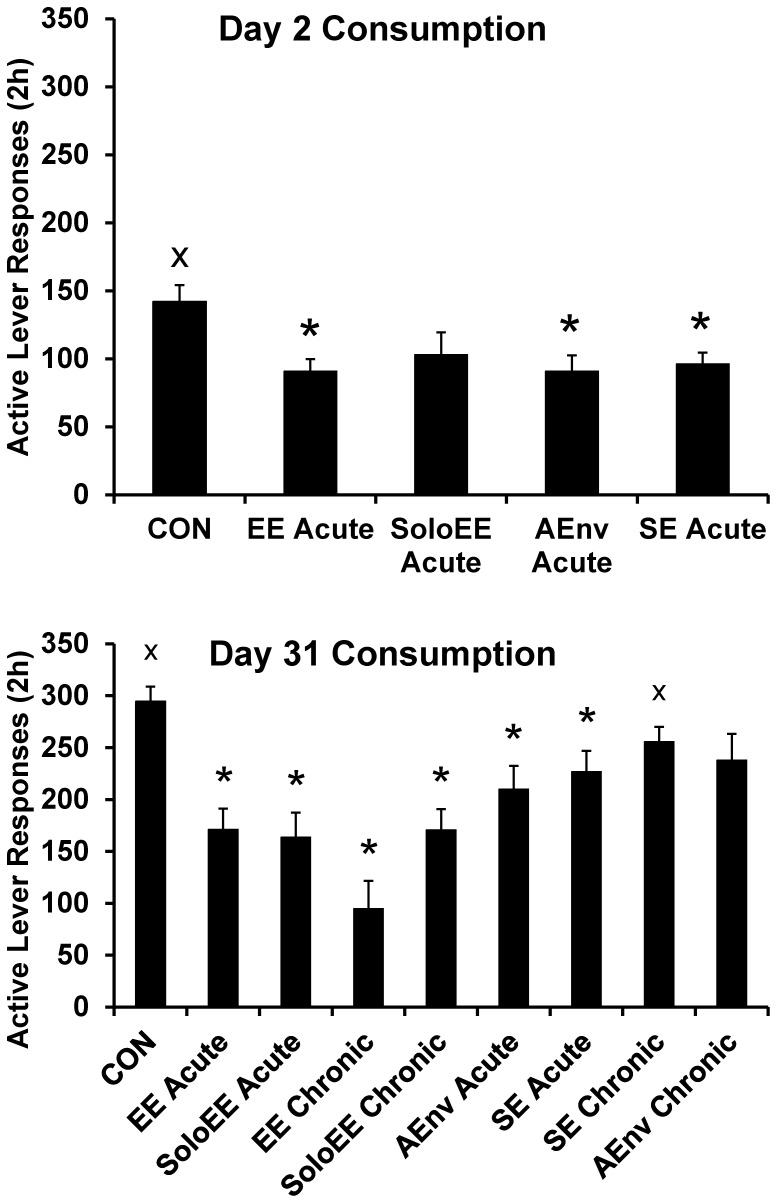
Sucrose Consumption the day following the Cue-reactivity test. All rats were housed in CON conditions following the Cue-reactivity test. * indicates significant difference from CON group and x indicates significant difference from EE Acute group, p<0.05.

For Day 2 responding there was a significant effect of MANIPULATION for active lever responses (F(4,55) = 3.3) and photobeam breaks (F(4,55) = 6.4), both p<0.05. For Day 31 responding there was a significant effect of MANIPULATION for active lever responses (F(8,103) = 10.2), inactive lever responses (F(8,103) = 2.5), and photobeam breaks (F(8,103) = 8.5), all p<0.05. Active lever responses and post-hoc tests results are presented in Figure 3. Data in Figure 3 are ranked according to the ranking of cue-reactivity responding in Figure 2. The effects of the various manipulations on inactive lever responses and photobeam breaks (with the exception of Day 2 inactive lever responses) were very similar to their effects on active lever responses. Means ± SEMS of inactive lever responses and photobeam breaks along with post-hoc tests are presented in Table 3.

**Figure 4:**
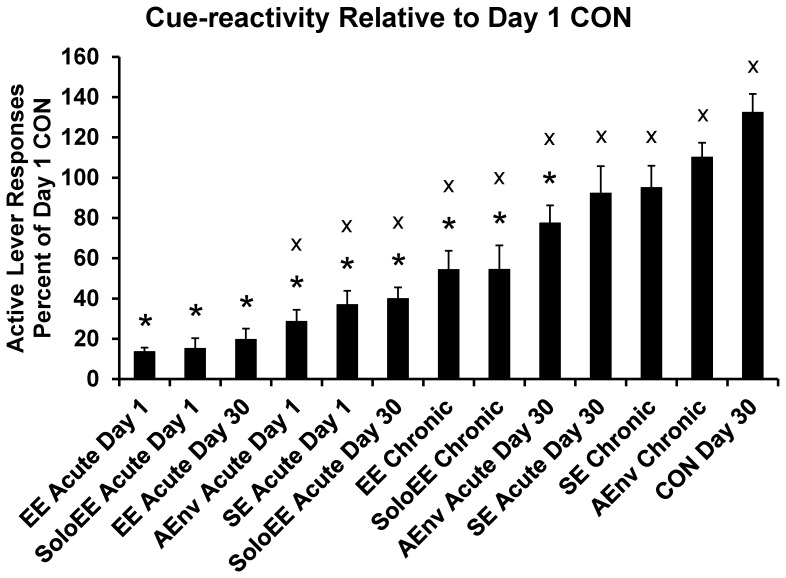
Cue-reactivity as a percent of Day 1 CON. Responding of a Day 30 group above 100% would suggest incubation of craving. Group data are presented ranked by group averages. * indicates significant difference from CON Day 30 group and x indicates significant difference from EE Acute Day 1 group, p<0.05.

Active lever responses as a percent of CON Day 1 responses were analyzed by ANOVA (13 levels including CON Day 30 but without CON Day 1). There was a significant effect of MANIPULATION F(12,148) = 19.9, p<0.001. These transformed data are presented in Figure 4 with the results of post-hoc tests. Data in Figure 4 are ranked from low to high. ANOVAs of Day 30 and Day 31 active lever responding of groups tested on Day 1 following an acute manipulation revealed no significant lingering effects of MANIPULATION (data not shown). That is, despite large effects of environmental manipulations prior to Day 1 testing, rats responded similar to CON rats one month later.

## Discussion

### Effects of Manipulations on Cue-reactivity

All acute manipulations except SE Acute Day 30 were effective at reducing sucrose cue-reactivity compared to CON rats. The EE and SoloEE chronic manipulations were also effective, but SE Chronic and AEnv chronic were not. It did appear that the non-significant SE manipulations (SE Acute Day 30 and SE Chronic) had some efficacy, however, those effects may have been masked by our statistical approach (see *Statisitical analyses*). Regardless, the most effective manipulation, by ranking, was the EE Acute condition. This was the case whether enrichment occurred before Day 1 or 30 cue-reactivity testing. In terms of statistical significance, EE Acute was more effective than AEnv Acute and SE Acute, but not SoloEE Acute, at the Day 1 time point (Figure 2). EE Acute was also more effective than all other treatments but SoloEE Acute at the Day 30 time point. As noted in the Results for most manipulations, a decrease in active lever responding was paralleled with a decrease in inactive lever responses and photobeam breaks (Table 2). This may indicate a general decrease in the incentive value of the sucrose-paired cues within the operant conditioning chamber.

### Effects of Manipulations on Sucrose Consumption

The present study was designed to optimize our ability to detect the effects of manipulations on sucrose cue-reactivity and since the acute manipulations were to be a one-night exposure, we chose to avoid another exposure prior to sucrose consumption testing (acute would no longer be acute). Despite this potential design limitation and the fact that cue-reactivity does not always predict self-administration (e.g. [9]), we were able to detect significant lingering effects of enrichment or novelty manipulations on sucrose consumption (Figure 3). For Day 2 testing, consumption was decreased to a similar extent across manipulations compared to CON rats, although the SoloEE manipulation failed to reach statistical significance. For Day 31, all manipulations but SE Chronic and AEnv Chronic reduced consumption; the greatest apparent decrease was in the EE *Chronic* group. Overall, the correlations between cue-reactivity and consumption for all rats were: Day 1 and Day 2 (n = 60) r = 0.57, Day 30 and Day 31 (n = 112), r = 0.56 (both p<0.001). Finally, as noted in the Results and above regarding cue-reactivity responding, a decrease in active lever responding in the consumption test was paralleled with a decrease in inactive lever responses and photobeam breaks (Table 3). Along with the decrease in overall responding during the cue-reactivity test and decreased active lever responding for sucrose during the consumption test, this indicates a general decrease in the incentive value of not only the operant conditioning chamber and sucrose-paired cues, but of sucrose as well.

### Proposed Mechanisms for EE Effects on Motivated Behaviors

EE has been shown to function as a natural reinforcer [14], as has novelty [15]. From a behavioral analysis perspective, exposure to enrichment or novelty may then create a contrast [16] such that when rats are subsequently allowed to respond for the sucrose-paired cue, they do not find it as reinforcing as the enriched or novel context from where they just arrived. We are still speculative regarding the actual mechanism of the EE effects in the present study. Yet, if our EE has reinforcing properties, our findings could complement other findings regarding alternative reinforcement effects in animal models of addiction. For example, wheel running access reduces cocaine cue-reactivity in rats [17] and access to alternative reinforcement during extinction accelerates extinction responding [18]. In the present study, the alternative reinforcement occurred in a context other than the operant conditioning chamber, expanding the conditions under which alternative reinforcement might alter operant responding.

Differing from this reinforcement hypothesis, Solinas and colleagues have suggested that the addiction-reducing effects of EE may be due to anti-stress effects of EE [19]. Anti-stress effects like this have been somewhat examined in recent studies. For example, plasma levels of corticosterone have been found to be decreased after acute EE in rats with a history of cocaine self-administration [20]. However, in this same report levels of corticosterone were no different when comparing chronically isolated rats vs. rats that had chronic EE. This contrasts even more from a finding in chronic EE-housed rats of corticosterone levels *above* isolated controls [21]. Clearly more needs to be done to evaluate the potential impact of stress on the effects of environmental enrichment.

### Impact of EE Components on Sucrose Cue-reactivity and Consumption

While there are no straight-forward comparisons in the literature for the acute manipulations on food or drug self-administration, our EE Chronic effects are in the same direction as some previous studies. And even though not statistically significant, our SE Chronic trends are also similar to previous studies. For example, chronic EE rats self-administer less ethanol than isolated rats and chronic SE rats are somewhat in between isolated and EE in their intake [22]. Chronic EE and SE rats do not escalate their self-administration of a relatively low dose of cocaine compared to isolated rats [23]. Chronic EE (female) rats have lower break points for cocaine than isolated rats [24], although the overall baseline rate of responding is greater in isolated rats. Chronic EE and SE rats also self-administer a relatively low dose of amphetamine at a lower rate than isolated controls [25]. Sucrose self-administration results are less consistent. Bardo et al. found that chronic EE rats initially self-administer sucrose pellets at a higher rate than chronic SE and isolated rats [25], but chronic EE and SE rats consume less sucrose (from a bottle) than isolated rats [26]. In studies of the impact of EE on drug *seeking*, rats exposed to social housing are more reactive to cocaine-paired cues than EE rats, but less than isolated-housed rats [27]. Social-housed rats are less reactive to a sucrose-paired cue than isolated-housed rats, but more than EE rats [28].

In the present study, the SE rats responded somewhat (but not significantly) less for the sucrose cue or for sucrose than CON rats, but generally more than EE rats (either acute or chronic) (Figures 2 and 3). These results fit the general pattern of the findings from the studies just described. Clearly social interaction does not account for the EE effects we observed in the present study, yet social interaction consistently impacts reward seeking and taking across drug and food reinforcers. Cain et al. reported that social housing reduces responding for a novel visual stimulus in rats (again, not as much of an effect as with EE) [15]. Some aspect of the social situation, perhaps reinforcing play behavior [29], may alter the motivation of rats to respond for reinforcers (primary or conditioned) or novelty.The inclusion of the SoloEE and AEnv conditions in the present study was an effort to isolate environmental factors beyond social interaction that might contribute to the EE effect. From what we manipulated, we found that exposure to an enriched environment without social cohorts was sufficient to reduce sucrose cue-reactivity and taking. The SoloEE effects we report are, perhaps, the first of their kind and demonstrate that enrichment of the environment alone can have a large effect on motivation for sucrose. We also found that the acute switch to a novel environment (AEnv) was sufficient, but chronic exposure was not–although there was a slight (non-significant) decrease in cue-reactivity and consumption in the chronic group. For consumption testing this might have been due, ironically, to the novelty of switching from the chronic AEnv condition to CON housing for the 24 h between cue-reactivity testing and sucrose consumption testing. The AEnv findings corroborate findings from another study where exposure to novelty in, or just prior to entering, the operant conditioning chamber delays the acquisition of amphetamine self-administration [30]. In summary we found that, in most instances, all of the “components” of EE alone are sufficient to reduce sucrose cue-reactivity and consumption. However, the most effective manipulations were those having the EE context.

### Acute vs. Chronic Manipulations

Almost all studies with enrichment manipulations have animals enriched for several weeks prior to behavioral testing. Most relevant to the present study are findings of reduced cocaine seeking by rats following less than 24 h environmental enrichment [11], [13]. Similar to their findings, we observed a dramatic decrease in responding for a cue previously associated with self-administration following acute exposure to EE. Both previous authors questioned whether acute EE effects were mediated by the same neurobehavioral mechanisms as chronic EE. We agree that the acute and chronic effects can be dissociated in some instances. For example, aspects of the environment are likely habituated to over several weeks and this was probably the case with all of the chronic manipulations we used. The passage of time in enrichment could also lead to the development of behaviors that could mediate sucrose seeking and consumption. For example, we previously hypothesized that a reduction in sucrose seeking following one month of environmental enrichment might have been due to an enhanced learning ability [10].

With this in mind, an explanation for the hypothesized reinforcement contrast effects we report here could be rapid alterations in the activity/microstructure of neural systems including the nucleus accumbens and orbitofrontal cortex that are involved in tracking the current value of a reward [31], [32]. Longer-term changes in brain function could mediate some of the chronic effects. These changes may occur in brain regions including the orbitofrontal cortex and frontal cortex. For example, chronic EE rats show decreased impulsive behavior when responding for sucrose [33]. Impulsivity is generally attributed to changes in orbitofrontal and prefrontal cortex function [34], [35]. We hope to identify key regions and messenger systems in future studies.

### Enrichment Manipulations Block Incubation of Sucrose Craving

Authors of a recently published study on rats with a history of cocaine self-administration concluded that environmental enrichment is not effective at blocking the incubation of craving effect [36]. These findings contrasted from what we reported in 2008 regarding rats with a history of sucrose self-administration [10], and somewhat from a report of an EE-mediated attenuation in incubation of cocaine seeking in rats [37]. In our previous study we compared the responding of rats on both days 1 and 30 of forced abstinence. Rats that were exposed to environmental enrichment during the 29 days of forced abstinence between cue-reactivity tests responded at similar rates on both days 1 and 30 of forced abstinence [10]. Thiel et al. compared the responding of rats that received essentially “acute” EE prior to a Day 1 test with the responding of rats that received essentially “chronic” EE prior to a Day 21 test [36]. Responding was higher for the Day 21 vs. Day 1 rats. In the present study, we observed a similar effect–responding on Day 30 by the EE Chronic rats was significantly greater than the EE Acute Day 1 rats (Figure 4). However, EE Acute Day 30 rats’ responding did not differ from the EE Acute Day 1 rats’ responding (both approximately an 85% reduction in responding compared to their appropriate control group). Alone, these data indicate that incubation was not observed in the EE Acute Day 30 rats. In fact, when considered as a percent of CON Day 1 average responding, five of the eight Day 30 tested groups (all except the SE manipulations and AEnv Chronic) responded significantly less than the CON Day 30 group and seven of the eight groups (all but AEnv Chronic) responded less than the 100% (CON Day 1) benchmark (Figure 4). Inasmuch as the CON Day 30 group represents incubated responding, these findings could be interpreted to mean that incubation was blocked to some extent in nearly all of these groups.

At this point we can only speculate as to how manipulations such as EE block the incubation of craving. For example, the “blocking” of incubation in the chronic EE group could have been due to a blunting of the development of incubation, while the blocking of incubation in the Day 30 EE Acute group could have been due to an effect specific to the expression of incubation. An alternative explanation is that both effects could have been mediated in the same way by EE functioning as alternative reinforcement. This may be the parsimonious explanation at present. As previously noted [13], [37], EE effects are transient. Although the present study was not designed to assess the persistence of the manipulations, we were able to corroborate this observation by examining cue-reactivity and consumption responding of rats on Day 30 and 31 that had received an acute manipulation prior to Day 1. ANOVAs of active lever responding on Days 30 and 31 revealed no significant effect of MANIPULATION (data not shown). If the acute manipulations had specifically impaired the development of incubation this should not have been the case. Overall the transience of the EE and other manipulations supports the hypothesis presented above that these manipulations produce at least a short-lived change in the reinforcing efficacy of the self-administration environment. From a practical standpoint these details regarding methods and interpretation will be critical in the development of future studies on how EE affects reward-seeking behavior.

Finally, as noted above, one particularly intriguing finding in the present study was that while both Acute Day 30 and EE Chronic reduced cue-reactivity (blocking incubation as argued above), the Day 30 EE Acute manipulation apparently had a greater effect on cue-reactivity while the EE Chronic manipulation had a greater effect on sucrose consumption (Figures 2 and 3). Active lever responding was not statistically significant between groups (p = 0.029 with a Bonferroni-corrected alpha of p<0.0073), but a follow up comparison of number of sucrose deliveries indicated that the groups did significantly differ (p<0.0073; data not shown). It could be that the chronic exposure to EE produces some additional changes in motivation for sucrose. This effect may be especially important for understanding the role of the environment in not only food-seeking, but food-taking behaviors. We plan to investigate the potential differential effects of acute vs. chronic EE on brain activity (e.g. fos activation following a cue-reactivity test) as a way to integrate these findings about enrichment with what is known regarding the neurobiology of the incubation of craving [38].

### Summary and Conclusions

Environmental enrichment had a profound effect of reducing sucrose cue-reactivity and consumption in rats with a history of sucrose self-administration. In most cases, conspecific access, environmental complexity, and exposure to novelty alone were sufficient to reduce sucrose cue-reactivity and consumption. However, the most robust decreases in cue-reactivity and consumption were observed when rats were exposed to an enrichment context either with or without social cohorts.

Our findings provide focus for future study of factors that mediate reward-related behavioral changes following environmental enrichment. Findings from this and future studies could provide a framework for ways to reduce reward seeking and taking. For example, it appears from ours and other studies with EE that reward seeking may be reduced by altering the value of an “addict’s” environment. Future studies elucidating the neuronal mechanisms underlying acute and chronic EE effects on behavior could lead to novel pharmacological tools for reducing addiction behaviors.
